# Clinic of the Jefferson Medical College

**Published:** 1852-06

**Authors:** J. Aitken Meigs


### CLINICAL REPORTS.

*Clinic of the Jefferson Medical College. Service of* PROFESSOR DUNGLISON.

(Reported by J. AITKEN MEIGS, M. D.)

[Continued from p. 331.]

CASE III. The next case to which we call your attention aptly illustrates the physiological dependence of the various organs of the economy upon each other, and the reciprocal action which they consequently exert.

We have elsewhere considered, somewhat in detail, the highly interesting subject of functional correlation. The harmony which so strikingly characterises the different actions of the human system, while in the healthy condition, we have seen to be the result of peculiar combinations of the two great sets of functions. The various correlations or combinations of these functions, are replete with interest to the physiologist; but it is not until they are applied as guides in the elucidation of pathological phenomena, that their real magnitude and importance become evident. The disturbing influence of disease, not unfrequently reveals to us an intimacy of connection, between certain portions of the body,—of which we had previously not the slightest conception. Hence we are occasionally called upon to observe how an apparently trifling derangement of one organ may be reflected, as it were, upon another; and with such force, moreover, as to light up in the latter a morbid action whose intensity and virulence, are with difficulty controlled. The latter of these abnormal actions being the stronger of the two, sometimes so masks the former, as to render the detection of the true cause of the mischief, and the formation of a correct diagnosis, extremely difficult.

In the patient whose symptoms we are about briefly to investigate in your presence, this principle is so prominently exemplified, that on account of this feature alone, we have been induced to bring him before you for examination and study; although his disease, in its general characters, differs but little, if at all, from the two preceding cases.

Mr. R. æt. 40, is very much troubled with violent pain in the heart, coming on at intervals, and accompanied with strong palpitation. He is not only incapable of prolonged exertion, in consequence of the annoying and dangerous impetus given thereby to the heart's action, but is compelled to avoid all unusual exercise, however temporary in duration, so that in ascending a flight of stairs at an accelerated pace, he is frequently obliged to stop and seize upon the bannisters for support. Notwithstanding these symptoms, however, auscultation shows no departure from the ordinary, healthy, cardiac sounds; a slight diminution in power, only, can be detected. The impulse is somewhat weakened.

He has never been affected with rheumatism.

There is tenderness at the epigastric centre; and he complains, furthermore, of a sensation of nausea, coming on shortly after each meal,—especially is this the case after the ingestion of fatty substances. The bowels are obstinately constipated.

Gastric disturbance was the first symptom of disordered health that attracted the notice of the patient. Soon the small intestines, and finally the entire alimentary track, became implicated. That the stomach is still in an unhealthy condition, is shown by the tenderness manifested when pressure is made over that organ. Physiology teaches us that fatty substances undergo no action in the stomach; but in the duodenum, form with the alkalies furnished by the bile and pancreatic juice, an emulsion which is gradually absorbed. Now, as you have already heard, the patient suffers most while in the act of digesting fatty or oily matters taken with his food. The application of a physiological fact, thus furnishes us, in this case, with proof that the small intestines are involved in the same morbid action. The extreme costiveness is a sufficient indication of a highly torpid condition of the colon.

We have here to contend, then, with two sets of symptoms: the one affecting the digestive apparatus, and arising mainly, perhaps, from faulty innervation of the intestinal tube; the other, affecting the heart, and dependent for existence on the former. To the first probably belongs the priority, in point of occurrence, and to it we must principally direct our treatment.

We have, thus, a well-defined example of an unhealthy action, originating in the digestive apparatus, steadily increasing in intensity to a certain point, and then suddenly reflected upon the circulatory system to the manifest disturbance of the whole economy. So, too, in hysterical females, we are often called upon to combat symptoms of functional derangement of the heart, evidently resulting from some uterine affection.

The indications for treatment in this case are very simple. We must obviate constipation—itself no trifling auxiliary in the production of heart disease—and develope the nervous sensibility of the intestinal canal. By arousing the digestive tube from its lethargy, we are in hopes that the cardiac symptoms,—which are the results of this lethargy,—will disappear as the cause is removed, and without further trouble.

We shall direct the following prescription, in which are combined a tonic and a saline cathartic, as in the Cheltenham water; and which, under a protracted use, is as well adapted for developing the sensibility of the intestinal tube, as any combination with which I am acquainted.

| | | |
|---|---|---|
| ℞. | Magnesiæ Sulphat. | ℥j. |
| | Potassæ Bitart. | ʒj. |
| | Ferri Sulphat. | ℈ss. |
| | Aquæ | Oj. |

M. A wine-glassful to be taken every morning before breakfast.

He will make use of an animal diet, and, in general terms, his food should be plain, nutritious and easy of digestion. Such articles as tea and coffee, fresh bread, the succulent vegetables, pastry, &c., or, in short, whatever he has known to disagree with him, must be carefully avoided.

Constrasting strongly with the cases which have just occupied our attention are the two which we now bring before you for examination and study. They are excellent examples of organic cardiac disease. In the one case there is hypertrophy of the left ventricle, with mitral regurgitation; in the other, hypertrophy of the same ventricle with, perhaps, semilunar regurgitation.

Let us consider them separately.

Case V. Mr. C——, æt. 40, of a robust, plethoric habit, and sanguine temperament, has been kind enough to present himself here, that his case may be investigated in your presence. About six weeks ago, he began to be aware of a sensation of unusual heat and tension behind the sternum, accompanied with an increase in the frequency and force of the heart's action. The impulse is daily becoming more violent and troublesome ; and he is beginning to suffer from dyspnœa, especially when the action of the heart is hurried by mental excitement or undue exertion.

Auscultation betrays a highly exaggerated impulse, with great obscurity of the cardial sounds,—so great indeed as to require close attention to distinguish them. Moreover, if you listen attentively, you will hear, synchronously with the systole of the heart, a rough, blowing sound—the "*bruit de souffle ou de soufflet*" of the French,—the "blowing or bellows-murmur" of the English writers. Now, this is an abnormous sound, demanding an inquiry into the accompanying phenomena ; for although not always of absolutely dangerous import, it is generally indicative of some structural lesion of the interior of the organ. The abnormal or exocardial heart-sounds, which are the consequence of morbid alterations exterior to the organ, are more or less frictional in character, and hence are usually readily distinguished from the endocardial, which are 'blowing.'

Endocardial sounds, or, to give them their generic title, "bellows sounds or murmurs," are the result of vibrations caused by columns of blood in their passage through the heart ; and you may comprehend that whatever has a tendency to affect these sanguineous currents, and change the healthy vibrations, may be the cause of such murmurs. Hence "blowing sounds" are frequently heard in anæmic persons—as in chlorotic females. Loss of blood may also occasion these murmurs ; and accordingly they are heard in persons exhausted and prostrated by hemorrhage.

In the majority of cases, however, in which the bellows sound occurs, it is symptomatic of morbid alteration of the valvular apparatus of the heart. Thus there may be fibrinous deposits upon and around the valves ; they may become cartilaginous, or ossified, or covered with warty excrescences, &c., giving rise to the different modifications or varieties of the murmur, known by the epithets *rasping*, *filing*, *sawing*, &c.

The general phenomena of this case lead to the inference, that there is valvular disease; and we must, accordingly, push our investigations further, in order to learn the seat of the mischief—whether it be at the aortic orifice, in the valves of the pulmonary artery, in the mitral or tricuspid valves, or in the auriculo-ventricular openings.

A little reflection will exhibit, that this can be approximated by ascertaining the exact spot in the præcordial region where the sound is heard the loudest, the direction in which it is transmitted farthest, and the time of occurrence, as compared with the systole or diastole of the heart.

Now, in derangement of the semi-lunar valves, the endocardial murmur is most distinctly heard near the third costal cartilage. When those of the aorta are in fault, the sound is transmitted upwards between the second and third ribs of the right side, following the course of the aorta; and when those of the pulmonary artery are affected, the sound extends to the left side, following the route of that artery. The place in which the murmur is heard, then, and the direction in which it is carried, furnishes us with the elements of diagnosis as to the valves specially implicated.

[This was elucidated by a diagram of a section of the heart figured on the black board.]

In this patient the murmur is heard much more loudly below and somewhat to the right of the left nipple, than at any other portion of the cardiac region, and is distinctly prolonged towards the apex of the organ.

In consideration of the aids thus afforded by auscultation, we have no hesitation, then, in concluding that we have here to contend with disease of the mitral valve. The tricuspid murmur to be sure, is heard loudest at about the same spot; but it is not so markedly directed towards the apex of the heart; and furthermore the pulse is generally not so much affected, and according to some, there is no accompanying "*fremissement cataire*" or purring-of-cat sound,—phenomena, which are the reverse of those presented here.

Case IV. David F——, æt. 35; colored; formerly a fisherman, now a bar-tender; has had symptoms of heart disease for the last five years. He complains of severe pain at the præ-

cordium, and is much disturbed by the palpitation and turbulent movements of his heart. The dulness on percussing the region of the heart is greater and more extended than usual; and to the ear of the ausculter is conveyed a clear and distinct bellows murmur, immediately following the impulse. The impulse is exceedingly strong and powerful; so forcible, indeed, that the head, in ausculting his chest, is seen by the class to be elevated by each systolic effort.

The pulse is very strong, not hurried, but having the jerking character which so often accompanies endocardial affections.

The strength and undue diffusion of the impulse, coupled with diminished clearness of the systolic sound,—facts presented by the case in consideration,—are indications of a hypertrophic condition of the left ventricle. Hypertrophy of the ventricles is a very common accompaniment of valvular disease. It is frequently, indeed, the effect of the difficult transmission of blood, owing to morbid changes in the valves; and the blowing murmur, in this instance, shows us that we have to contend with the complication in question.

The character of the pulse, the location of the bellows sound, and the concomitant symptoms, induce in our mind the belief that there is some deposition upon or imperfection of the semilunar valves at the mouth of the aorta, thus permitting a portion of the blood, after every contraction of the ventricle, to regurgitate from the aorta. Yet, contrary to the usual rule in these cases, which has been referred to in the course of this lecture, we have the sound transmitted in the direction of, and heard most strongly over the apex of the heart, instead of at its base. This anomaly complicates the diagnosis as to the nature and seat of the cardiac lesion; and we might be induced to consider the mitral valve in fault. But if such were the case, how could we explain the bellows sound occurring so distinctly after the impulse, and taking the place of the ordinary second sound of the heart? The phenomena appeared at first sight to sanction the view mentioned by some, that the impulse is synchronous with the first stage of the diastole of the organ, or with what they consider the contraction of the auricles; but the synchronous occurrence of the impulse and of the pulse at the wrist is a fatal objection to this view, inasmuch as the latter phenomenon—the pulse—is unquestionably produced by the systolic impulse on the column of blood contained in the arterial vessels. Moreover, as was explained to you elsewhere, there is but a slight action of the auricles during the first stage of ventricular dilatation; the real contraction of their muscular parietes being concerned in sending the last portions of blood into the ventricles at the termination of the interval of repose, and this auricular contraction being rapidly propagated through the muscular structure of the ventricles to the apex of the heart; so that the auricular and the ventricular systole form part, as it were, of but one movement, so progressively and intimately are they associated; and hence we can understand the common combination of hypertrophy of the auricles with hypertrophy of the ventricles.

[Here the physiology of a series of the heart's actions was explained by a diagram on the black board.]

In the case before us it will be borne in mind, that the bellows murmur was as distinctly succeeding to, and separable from the impulse of the heart against the chest, as is the normal second sound from the first sound of the organ. If, therefore, the impulse is occasioned by the combined auriculo-ventricular contraction, as it would seem to be, although the matter cannot be regarded as demonstrated, then the bellows murmur in this case must be owing either to the regurgitation of the blood through the aortic semilunar valves, or to some disease of the mitral valves, which obstructs the regular course of the blood from the auricle into the ventricle; and the fact that the sound is more distinctly heard as the ear approaches the apex, is confirmatory of this view of the subject. If, however, so much morbid alteration of the mitral valve is present as to account for the phenomena, it is not easy to understand, why, during the systole of the organ, there should not be decided physical evidences of mitral regurgitation; and, on the other hand, if the morbid change be in the semilunar valves of the aorta, the only hypothesis we can suggest for the murmur being more distinctly heard near the apex than the base of the heart is the existence of physical circumstances in the special formation of the chest, that may, in some manner, modify the ordinary transmission of the physical signs to the ear of the observer.

As to the therapeutics of these two cases, and indeed of all organic affections of the heart, obviously but little can be done. They are structural changes, which cannot be reached by any system of medication. Certainly, in the whole domain of materia medica, we cannot find an article which exerts a positively curative influence upon the diseased structure ; and we are consequently obliged to fall back upon general principles, which, although palliative in character, are oftentimes found to be productive of no little good. When within certain limits, such disease is not always speedily or necessarily fatal, and life may often be prolonged with comparatively little discomfort, by a judicious regulation of the habits and circumstances of the patient.

The great and troublesome palpitation in the first case, we shall endeavor to ameliorate by the exhibition of tinctura digitalis, given to the extent of twenty or thirty drops in the course of the day, carefully watching its operation, and arresting it when its sedative agency is exerted beyond the necessary extent. In the other case, we shall make use of no medicine whatever, but depend upon an exclusively hygienic treatment. Both patients we shall direct to live with the utmost abstinence, taking readily digestible food in quantity merely sufficient to support life. They must avoid stimuli of all kinds ; observe strictly the antiphlogistic regimen ; and maintain, at all times, quietude of mind and body, as far as this is practicable. In taking exercise care must be had to avoid every cause of undue excitement. They should not run, nor walk rapidly, nor climb steep ascents, nor lift heavy weights, nor, in short, make use of any unusual effort. Attention should also be directed to the digestive organs, derangement of which so constantly reacts on the cardiac functions. Nothing further shall be done at present than to direct them, in common with the others, to return a week hence, and report progress.

